# Hysterosalpingographic findings among Ghanaian women undergoing infertility work-up: a study at the Korle-Bu Teaching Hospital

**DOI:** 10.1186/s40738-015-0001-6

**Published:** 2015-06-04

**Authors:** Benard Ohene Botwe, Kwabena Bamfo-Quaicoe, Edem Hunu, Samuel Anim-Sampong

**Affiliations:** Department of Radiography, University Ghana School of Biomedical and Allied Health Sciences, College of Health Sciences, P.O. Box KB 143, Korle Bu, Accra, Ghana

**Keywords:** Hysterosalpingography, Hydrosalpinx, Fibroids, Tubal blockage, Female infertility

## Abstract

**Background:**

Hysterosalpingography (HSG) is one of the commonly used radiological modalities for investigating infertility in women. In developing countries such as Ghana it remains the main tool for investigating some of the underlying causes of female infertility. The purpose of this study was to determine the predominance of various hysterosalpingographic results in patients that went through infertility work-up at the Korle-Bu Teaching Hospital between January 2011 and December, 2014. This is to help plan for preventive measures for the communities.

**Results:**

This study collected retrospectively, 1140 consecutive radiologic reports from January 2011 to December, 2014 from the Department of Radiology, Korle-Bu Teaching Hospital. These reports were reviewed and diagnoses made were evaluated with Microsoft Excel. Secondary infertility was 52.4 % prevalent and primary infertility was 41 % prevalent. While 453 (39.7 %) patients presented with normal findings, 153 (13.4 %) had hydrosalpinx, 498 (43.6 %) had tubal blockage, and 290 (25.4 %) had fibroids. Also there were 10 (0.9 %) cases of patulous cervices, 8 (0.8 %) cases of uterine synechiae, 62 (5.4 %) of irregular uterine outline, 15 (1.3 %) of irregular cervical outline, 3 (0.3 %) of arcuate uteri, and 5 (0.4 %) cases of retroverted uteri.

**Conclusion:**

Tubal blockage which presented in 43.6 % of patients was the most common abnormal finding at HSG examinations carried out between January 2011 and December, 2014 at the KBTH. This was followed by fibroids with 25.4 % and hydrosalpinx with 13.4 %. Factors such as chronic pelvic inflammatory disease or pelvic infection following STIs, mismanaged pregnancies and septic abortions, may have accounted for this since the majority of the women presented with secondary infertility.

## Background

The World Health Organisation (WHO) defines primary infertility as the inability of a couple to conceive within 2 years of exposure to the risk of pregnancy (i.e. sexually active, non-contracepting and non-lactating), while other literature define it under the same conditions but with respect to a 12-month time limit [1, 2]. Secondary fertility on the other hand is simply defined by the National Institute for Clinical Excellence (NICE) as the inability to conceive after a previous pregnancy [3]. Irrespective of the infertility type, the effect which plagues about 48.5 million couples around the world is very great especially in some African and Asian countries where women are expected to reproduce soon after marriage [4]. Failure leads to stigmatisation and marital instability. It is estimated that 15 % of all women experience primary or secondary infertility at one point in time in their reproductive life [5, 6]. As a result, women who present signs of infertility are made to undergo medical investigations in order to address any infertility problems they may have.

One of the most commonly used radiological modalities for investigating infertility in women is hysterosalpingography (HSG) [7, 8]. HSG is basically the radiographic evaluation of the uterine cavity and fallopian tubes by administering radio-opaque contrast medium into them [9, 10]. Its sensitivity and specificity are estimated at 65–81.41 % and 47.8–50 % respectively for tubal pathologies [11–13], and 92.1 and 85.7 % respectively for tube patency or tube blockage [14]. For peritubal adhesions the sensitivity and specificity are estimated at 35.5 and 81.3 % respectively [12].

Because HSG is a safe, readily available, relatively inexpensive, simple and rapid diagnostic test, it remains an important investigation in the management of infertility in women in Africa including Ghana [7]. The pathologies detected on HSG may include tubal blockage, peritubal adhesion, submucosal leiomyoma, endometrial polyp, endometrial carcinoma, synechiae and adenomyosis among others [15].

Previous studies on the outcome of HSG work-ups have presented different results regarding the prevalence of the findings. A study in Uganda [16] registered the major findings of HSG as bilateral tubal blockage (31.5 %), unilateral tubal blockage (6 %), hydrosalpinx (12.8 %), and tubal ligation (4.2 %). Others were fimbrail-end adhesions (17.3 %) and congenital abnormalities (1.6 %), while normal findings were 16.5 %. A Nigerian [17] study also found uterine fibroid (26 %) as the commonest pathology on HSG, followed by uterine adhesion (12 %), peritubal adhesion (10 %), tubal block (7 %) and hydrosalpinx (6 %)”. In Turkey, 21 % of the infertile women had one sided tubal occlusion and 12 % had bilateral tubal occlusion. Features for adnexal adhesion were also seen among 12 % of the infertile women [18]. These variations in the findings of HSG therefore call for an evaluation of what prevails in Ghana.

Consensus across literature also suggests that there is a higher prevalence rate of secondary infertility (as high as 38 %) as compared to 1–8 % for primary infertility in developed countries while, the opposite is true for Sub-saharan Africa [6, 19, 20]. The high secondary infertility prevalence in Sub-saharan Africa is suggested to be due to Sexually Transmitted Infections (STIs) and medical interventions carried out under non-sterile conditions particularly during delivery or induced abortion in certain parts of the region [21].

At the Radiology Department of the Korle-Bu Teaching Hospital (KBTH), which is the biggest hospital in Ghana, women turn-up for HSG examinations every working day with indications such as primary infertility, secondary infertility, recurrent spontaneous abortions, preoperative evaluation prior to myomectom and postoperative evaluation following tubal ligation among others. However, the predominance of the findings of the HSG work-ups have not been evaluated and documented. This retrospective review was therefore performed to assess the prevalence of HSG findings in patients that went through infertility work-up between January 2011 and December, 2014 at the KBTH.

## Method

A retrospective review of all conclusive radiological reports of 1140 infertile women who underwent HSG during their infertility work-up at KBTH between January 2011 and December 2014 was undertaken. Prior to the study ethical approval and permission to carry out the research work were respectively sought from the Ethics and Protocol Review Committee of the School of Allied Health Sciences, University of Ghana, and the Radiology Department of KBTH to enable access and review of available radiological reports retrieved from the Filing and Archiving Section of the Radiology Department. The reports that indicated no abnormalities (whether Mullerian or pathological) were separated from those that presented with abnormalities, while those that suggested abnormalities had the appropriate abnormality/abnormalities recorded and classified accordingly. Statistical Package for the Social Science (SPSS) version 18.0 used to analyse the data. The chi-square goodness of fit test tool of SPSS was used to test for association between some HSG findings and patient age. Descriptive analysis by way of frequencies and, graphs was the basis for drawing conclusions.

In accordance with ethical considerations of ensuring confidentiality and protection of patients’ privacy, patients’ identities were coded.

## Results

Following the study, 1140 conclusive radiological reports were reviewed. The ages of the infertile women ranged between 19 and 48 years, with a mean of 33.2 years (Table 1). The 30–34 years group presented the highest number of patients (*n =* 391, 34.3 %) submitting to infertility investigation by HSG. The least submissions were presented by the 15–19 years group (*n* = 2, 0.18 %) and 45–49 years (*n* = 19, 1.67 %)

**Table 1:** Age distribution of patients

Age range (years)	Frequency	Percent (%)
15–19	2	0.16
20–24	39	3.43
25–29	257	22.54
30–34	391	34.30
35–39	307	26.93
40–44	125	10.97
45–49	19	1.67
Total	1140	100.00

Mean age: 33.2 years

The distribution of the patients’ clinical histories and findings at HSG are presented in Tables 2 and 3 respectively.

**Table 2:** Distribution of patient history

History	Frequency	Percent (%)
Primary infertility	467	41.0
Secondary infertility	597	52.4
Myomectomy	37	3.2
Salpingectomy	18	1.6
Pregnancy termination	14	1.2
Cesarean section	7	0.6

**Table 3:** Distribution of findings at HSG

Findings	Frequency	Percent (%)
Normal findings	453	39.7
Abnormal findings		
Left tubal block	143	12.5
Right tubal block	121	10.6
Bilateral tubal block	234	20.5
Left hydrosalpinx	45	3.9
Right hydrosalpinx	47	4.1
Bilateral hydrosalpinx	61	5.4
Fibroids	290	25.4
Patulous cervix	10	0.9
Retroverted uterus	5	0.4
Uterine synechiae or Asherman’s syndrome	8	0.8
Irregular uterine outline	62	5.4
Irregular cervical outline	15	1.3
Arcuate uteri	3	0.3

284 (24.9 %) had multiple abnormalities

**Irregular cervical and uterine outlines, hydrosalpinx, tubal block, fibroids, patulous cervices, arcuate, uterine synechiae or Asherman’s syndrome were classified as abnormalities

**Fig. 1:**
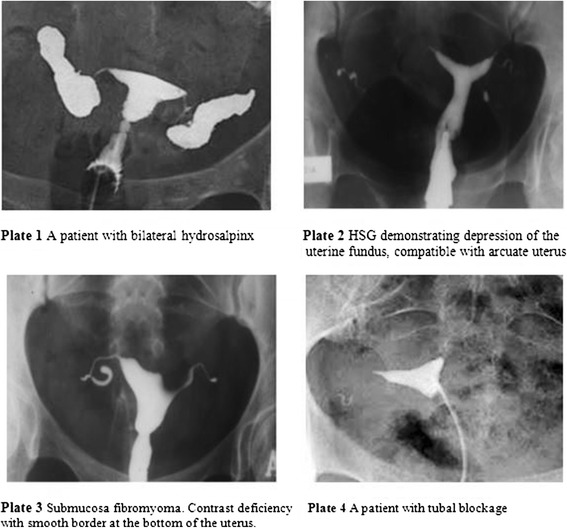
Sample images of HGS

Sample images (plates) of the abnormal HSG findings of patients presenting with bilateral hydrosapinx, arcuate uterus, submucosal fibromyoma, and tubal blockage are presented in Fig. 1.

**Fig. 2:**
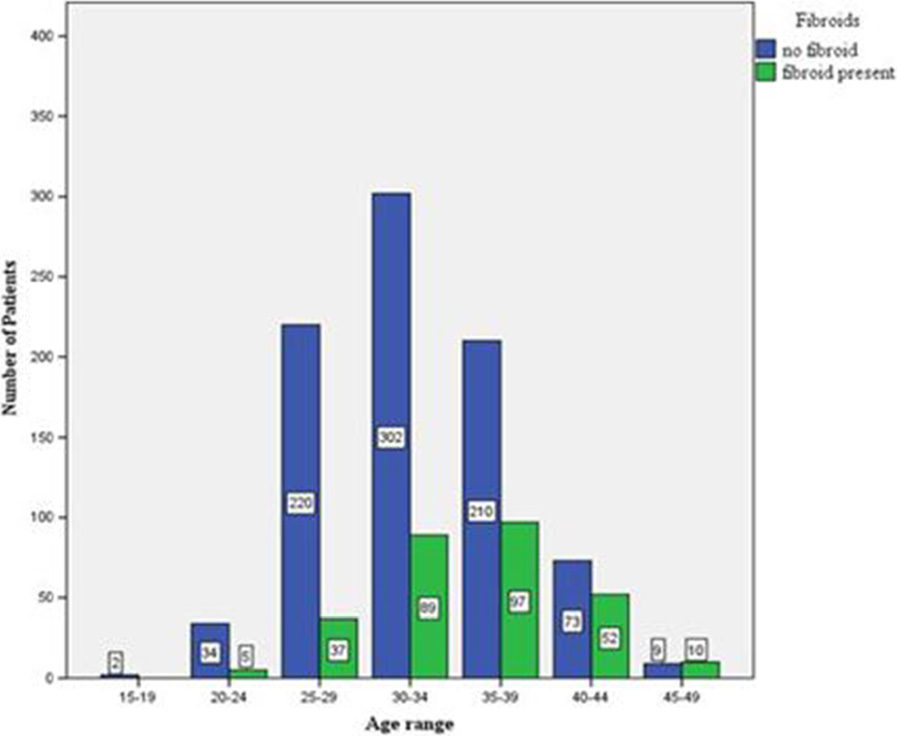
Association of age and fibroids

From Fig. 2, it was observed that as age advanced the number of patients with fibroids also increases but decreases after 40 years. This association was statistically significant [contingency coefficient (CC) = 0.21, *p* < 0.001].

**Fig. 3:**
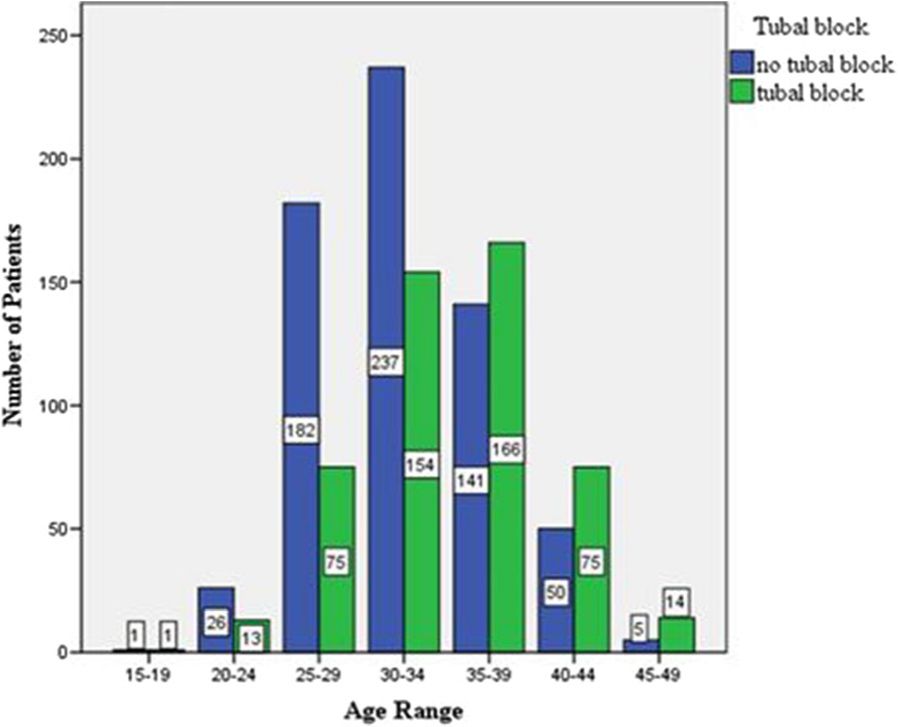
Association of age and tubal blockage

From Fig. 3, it was observed that the number of patients with tubal block also increased with advancing age. This trend reversed after 40 years. This association was statistically significant [contingency coefficient (CC) = 0.23, *p* < 0.001].

**Fig. 4:**
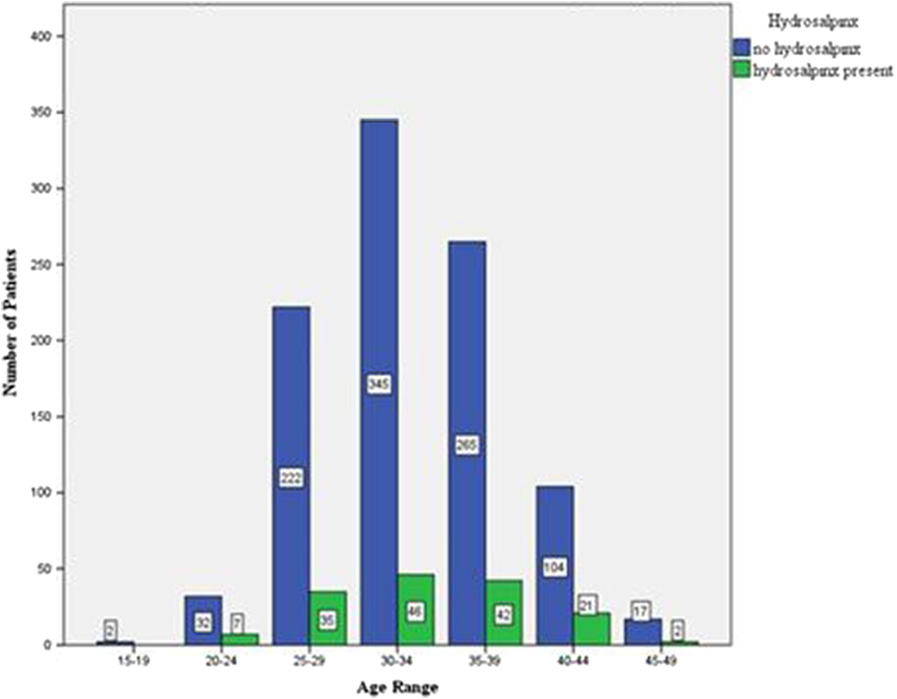
Association of age and hydrosalpinx

From Fig. 4, it was observed that the number of patients with hydrosalpinx increased gradually with age and peaked at the 30–34 age range. This began to dwindle down at 40 years. No evidence of association between patient age and the presence hydrosalpinx was observed as *p*-value was greater than 0.05 [χ^2^ *(11) =* 18.75, *p = 0.408*].

## Discussion

Infertility affects an estimated 48.5 million couples around the world [4]. Although the incidence of infertility varies in different parts of the world, in this study women presenting with secondary infertility (52.4 %) were higher than those presenting with primary infertility (41 %) which is consistent with other studies by [6, 20, 22], but deviates from studies carried out in Iran [23]. This outcome is also consistent with the general opinion that secondary infertility in women is of higher prevalence in developing countries than primary infertility while the opposite is true for developed ones. It is suggested that the higher prevalence of secondary infertility over primary infertility in Sub-Saharan Africa such as Ghana is attributed to post-abortal sepsis, puerperal sepsis, and PID resulting from STIs especially [21].

The age distribution of the patients that subjected to HSG were between 19 and 48 years, while the 30–34 years group had the highest number of patients. In African setting women marry early; therefore the fact that majority of the patients were aged within their early 30s is suggestive that a majority may have tried severally to have babies for a long while without results and desperately needed medical interventions before it becomes too late.

Statistically, 39.7 % of the infertile women in this study had normal findings, indicating that the cause of their inability to conceive could be attributed in part to their male counterparts. On the contrary, the cause could be on the womens’ part due detection failures since HSG lacks some sensitivity. This finding is higher than the 16.6, 29.4 and 36 % recorded in Uganda [16], Nigeria [20] and Ethiopia [22] respectively. It is however, lesser than the 55 % as reported in Switzerland [8]. It also worth noting that in addition to its diagnostic value, HSG is also used for therapeutic purposes to unblock the blocked fallopian tubes and that may have resulted in some of the normal findings.

Tubal block which typically prevents successful passage of an egg to the sperm, or fertilized egg to the uterus, and manifests on HSG as an abrupt cutoff of contrast material with non opacification of the more distal fallopian tube, was found to be main structural cause of infertility in women per HSG findings at KBTH between 2011 and 2014. The prevalence of tubal blockage determined at 43.7 %, (10.6 % for right, 12.5 % for left and 20.5 % for bilateral occlusion) was less than the 23.3 % as reported [24]. It must be mentioned that spasm could have accounted for some of the tubal blockages. The tubal blockage observed in this study was found to increase with age but decreases at the age 40 and above. Bilateral or unilateral hydrosalpinx which also recorded a prevalence of 13.4 %, together with tubal blockage which is a tubal factor, accounted for 57 % of the abnormal findings. These findings are a reflection of high prevalence rate of pelvic inflammatory diseases in Ghana.

The common uterine pathology in this study was the presence of fibroids (also called myomas) estimated at 25.4 %. Infertility is rarely caused by myomas (in less than 10 % of infertilities), but when it is, it is associated with a submucous myoma or a markedly distorted, enlarged endometrial cavity that interferes with normal implantation or with sperm transportation [25, 26]. Therefore HSG is of great value in evaluation of uterine cavity and fallopian tubes patency when planning for myomectomy, as were the indications of 3.2 % of the patients. The study found that as age increases the likelihood of fibroid formation is high. This trend however decreases at age 40 and below. There is the need to investigate why fibroid appears to decrease in people above 40 years old.

Of the many congenital uterine anomalies, only 3 (0.2 %) arcuate uteri were diagnosed. This is lesser than all the rates reported for these anomalies in all reviewed works. The closest was 1.6 % reported in Uganda [16], which is 8 times higher than that recorded in this study, suggesting a lower incidence at KBTH, and probably in the country. Though arcuate uteri were the only CUA reported in this study, they are the least known for adverse effects such as miscarriage, pre-term delivery, and fetal mal-presentation at delivery [27].

There were 0.8 % *(n =* 8) patients with uterine synechiae or Asherman’s Syndrome, a condition characterised by adhesions and/or fibrosis of the endometrium most often associated with dilation and curettage of the intrauterine cavity [28]. This is far less than any of the rates recorded by reviewed literature. The closet value to this is the 1.5 % reported in the literature [29] at HSG.

About 0.4 % cases of retroverted uteri were also recorded. According to published literature [28], retroverted uteri even have marginally higher clinical pregnancy and implantation rates than anteverted uteri which were not encountered in this study.

Patulous cervices were found in 10 (0.9 %) patients. This can be attributed to injury (due to abortion, curettage, conisation and dilatation, prior pregnancy, or an ectopic pregnancy) or by congenital causes (30). The Nigerian study [20] found this to be 1 case out of 272 (0.37 %) which is comparable to the finding in this study.

Irregular uterine outlines were recorded in 62 (5.4 %) patients and irregular cervical outlines were recorded in 15 (1.3 %) of patients. This may be due to endometritis following PID, post abortal or post-partum infections or some medical instrumentation [24]. This distortion of uterine cavity usually results in infertility due to failure of embryo implantation or spontaneous abortion [28].

## Conclusion

The most common structural cause of infertility in the women who underwent HSG work-out at the KBTH between January 2011 and December, 2014, was tubal blockage (43.6 %). Factors such as chronic pelvic inflammatory disease or pelvic infection following STIs, mismanaged pregnancies and septic abortions, may have accounted for this since the majority of the women presented with secondary infertility. This was followed by fibroids with 25.4 % and hydrosalpinx with 13.4 %. Other findings were irregular uterine outline 5.4 %, irregular cervical outline 1.3 %, patulous cervix 0.9 %, retroverted uterus 0.4 %, uterine synechiae or Asherman’s syndrome 0.8 %, and arcuate uteri 0.3 %. Primary prevention of reproductive tract infections is therefore very vital to reducing the unacceptable high incidence of infertility in our environment.

## Abbreviations

HSG: Hysterosalpingography
STIs: Sexually Transmitted Infections
PID: Pelvic inflammatory disease
CUA: Congenital *uterine* anomalies
